# A study of the prostate, androgens and sexual activity of male rats

**DOI:** 10.1186/1477-7827-5-11

**Published:** 2007-03-16

**Authors:** Maria Elena Hernandez, Abraham Soto-Cid, Gonzalo E Aranda-Abreu, Rosaura Díaz, Fausto Rojas, Luis I Garcia, Rebeca Toledo, Jorge Manzo

**Affiliations:** 1 Instituto de Neuroetologia, Universidad Veracruzana, Xalapa, Ver., Mexico; 2 Facultad de Quimica Farmaceutica Biologica, Universidad Veracruzana, Xalapa, Ver., Mexico

## Abstract

**Background:**

The prostate is a sexual gland that produces important substances for the potency of sperm to fertilize eggs within the female reproductive tract, and is under complex endocrine control. Taking advantage of the peculiar behavioral pattern of copulating male rats, we developed experimental paradigms to determine the influence of sexual behavior on the level of serum testosterone, prostate androgen receptors, and mRNA for androgen receptors in male rats displaying up to four consecutive ejaculations.

**Methods:**

The effect of four consecutive ejaculations was investigated by determining levels of (i) testosterone in serum by solid phase RIA, (ii) androgen receptors at the ventral prostate with Western Blots, and (iii) androgen receptors-mRNA with RT-PCR. Data were analyzed with a one-way ANOVA followed by a *post hoc *application of Dunnett's test if required.

**Results:**

The constant execution of sexual behavior did not produce any change in the weight of the ventral prostate. Serum testosterone increased after the second ejaculation, and remained elevated even after four ejaculations. The androgen receptor at the ventral prostate was higher after the first to third ejaculations, but returned suddenly to baseline levels after the fourth ejaculation. The level of mRNA increased after the first ejaculation, continued to increase after the second, and reached the highest peak after the third ejaculation; however, it returned suddenly to baseline levels after the fourth ejaculation.

**Conclusion:**

Four consecutive ejaculations by sexually experienced male rats had important effects on the physiological responses of the ventral prostate. Fast responses were induced as a result of sexual behavior that involved an increase and decrease in androgen receptors after one and four ejaculations, respectively. However, a progressive response was observed in the elevation of mRNA for androgen receptors, which also showed a fast decrease after four ejaculations. All of these changes with the prostate gland occurred in the presence of a sustained elevation of testosterone in the serum that started after two ejaculations. A consideration of these fast-induced changes suggests that the nerve supply plays a key role in prostate physiology during the sexual behavior of male rats.

## Background

The prostate is a sexual gland that produces important substances for the potency of sperm to fertilize eggs within the female reproductive tract. In order to accomplish this task, the prostate gland is finely regulated by neural and hormonal mechanisms, and possesses a complex histological organization. It is located at the proximal region of the urethra, the prostatic urethra, as a well-defined globular gland showing two clearly distinctive regions, the dorsolateral prostate and the bilobulated ventral prostate. Histological examination of the gland reveals that the two regions are organized into several alveoli surrounded by a stroma area. Each alveolus is arranged with a secretory epithelium involved in the synthesis of prostatic secretions.

The function of the prostate is under complex endocrine control. We showed previously that mating-induced release of prolactin (PRL) was important for the proper function of the epithelial cells, with a precise mechanism controlling its release since constant elevated levels of PRL caused detrimental effects on both the gland and male sexual behavior [1]. On the other hand, steroids have long been considered an essential part of prostate endocrinology, with androgens regarded as the etiology of prostate cancer following the early work of Charles B. Huggins. Androgen deprivation is therefore one of the most common therapies used today [2]. However, it is known that androgens play a key role in the adult male for the maintenance of several vital functions of the body, including those necessary for sexual behavior and reproduction [3-7]. Androgens have a strong impact on the prostate in adult subjects in terms of the maintenance of its morphology and secretory activity [8-10], with the ventral prostate being the main area that responds to androgen stimulation [11-13]. Additionally, androgens have a dual role in stimulating proliferation and inhibiting death of epithelial cells [14], which suggests the existence of a mechanism to avoid the pathological growth of the gland. Prostate responses to androgens are mediated by the wide distribution of androgen receptors (AR) in epithelial cells, smooth muscle, and stroma cells [15,16]. It has been shown that the complex androgen-AR is translocated to the nucleus to regulate gene transcription [17], and that androgens produce an increase in both the level and half-life of AR [18]. However, the modulation of androgen and AR levels in the prostate of healthy subjects with a constant sexual life remains unknown.

The sexual behavior of male rats follows a well-known stereotyped pattern. Males display several mounts and intromissions before ejaculation, and all of these events occur recurrently in such a way that a male can achieve several ejaculatory events in a single sexual encounter. Taking advantage of this behavioral pattern, we developed experimental paradigms to determine the influence of sexual behavior on the level of serum testosterone, prostate androgen receptors, and mRNA for androgen receptors in male rats displaying up to four consecutive ejaculations.

## Methods

### Subjects and housing

Sexually experienced Wistar male rats (250–300 g/bw) were used in this investigation. Males were kept in a room with an inverted light-dark cycle (12-12 hr, lights off at 0800 hrs). Every rat was manipulated daily to promote habituation to experimental contact and the environment in order to minimize stress. Ovariectomized females were used and their sexual receptivity was induced with steroids dissolved in sesame oil. Accordingly, subcutaneous injections of estradiol benzoate (10 μg; Sigma-Aldrich Quimica, Mexico) and then progesterone (2 mg; Sigma-Aldrich Quimica, Mexico) were administered 48 and 4 hrs before tests, respectively. Rats were housed in plastic cages (50 × 30 × 20 cm) containing wood chip bedding, and were placed in rooms were under a controlled temperature (22 ± 2°C), with food (Harlan Mexico rodent chow) and water available *ad libitum*. Every surgical intervention and manipulation of rats was guided by the Society for Neuroscience Policy on the Use of Animals in Neuroscience Research.

### Experimental design and determination of testosterone serum level

The experimental paradigm of this study was used to determine the effect of consecutive ejaculations (up to 4) on the level of serum testosterone and the expression of androgen receptors at the ventral prostate. Males were prepared for blood collection two days before the beginning of tests, and females were treated appropriately to induce a state of sexual receptivity. Blood collection was obtained from the jugular vein as described previously [1]. Experiments were carried out under red light between 12:00 and 16:00 hrs as follows: A male rat was taken from its cage, placed in a Plexiglas arena (60 cm diameter × 60 cm high), and five minutes later a receptive female was introduced. Parameters detailing the sexual behavior of the male were recorded immediately [19]; only the Hit Rate parameter is shown in the results. It represents a proportion obtained after the computation of the number of intromissions divided by the sum of the number of intromissions and the number of mounts [20], and is a good indicator of the appropriate execution of a male's sexual behavior.

The number of consecutive ejaculations marked the assigned group of the animal. Males from group E1 (n = 8) were allowed to ejaculate once, and then a post-ejaculatory blood sample was obtained. Group E2 males (n = 8) were allowed to ejaculate twice, group E3 males (n = 8) were allowed three ejaculations, and group E4 males (n = 8) were allowed four ejaculations, with post-ejaculatory blood samples being obtained after the final ejaculatory event of each male. Each blood sample was allowed to coagulate for 20 min, centrifuged for 10 min (at 3,500 rpm), and the serum was collected and centrifuged again. The resulting serum was divided in 400-μl aliquots that were stored at -70°C until assayed for testosterone by solid phase RIA with I^125 ^[21] (sensitivity = 0.04 ng/ml; intra- and inter-assay coefficients of variations were 8.4 and 15.2, respectively). Additionally, the ventral prostate of each male was removed, weighed and frozen immediately in liquid nitrogen until analyzed.

### Determination of AR at the ventral prostate

Tissue from the ventral prostate (150 mg) was homogenized with 400 μl of Buffer "A" (HEPES 1 M pH 7.9, KCl 1 M, EDTA 0.5 M pH 8, PMSF 100 mM pH 8, and Protease Inhibitor Cocktail), and incubated for 20 min at 4°C. 25 μl of 10% NP-40 was then added during constant movement. The mixture was centrifuged (1 min at 10,000 rpm, 4°C) and the liquid phase (cytoplasmic) separated. The precipitated nuclear extract was re-suspended with 100 μl of Buffer "C" (HEPES 1 M pH 7.9, NaCl 4 M, EDTA 0.5 M pH 8, EGTA 10 mM pH 8, DTT 100 mM, PMSF 100 mM pH 8, and Protease Inhibitor Cocktail), and vortexed for 30 min at 4°C. 30-μl aliquots were separated and stored at -70°C until used. Chemicals were obtained from Sigma-Aldrich Quimica, Mexico.

The level of total protein was measured using the Bradford method [22]. Briefly, a standard curve was obtained with solutions containing 1, 2, 4, 8 and 12 μg/μl BSA, to which was added 180 μl of the Bradford solution (BioRad, Mexico). After a resting period of 5 min, the absorbance was read at a wavelength of 595 nm with a microplate spectrophotometer (BioRad 550). The samples were prepared with 20 μl of the nuclear extract diluted 1:20 with the Bradford solution. Linear regression analysis was performed with Prism 4 software (GraphPad Software Inc., San Diego, CA), and produced a correlation index ≥ 0.95.

The level of AR was measured using the Western Blot procedure [21]. Samples of 100 μl were prepared with 2% 2-β mercaptoethanol heated in a bain-marie for 5 min at 60°C. AR was separated using 10% polyacrilamide gel electrophoresis in the presence of SDS (SDS-PAGE; MiniProtean III, BioRad) at 100 V. The protein blot on the nitrocellulose membrane was performed for 2 hrs at 4°C with a current of 150 mA, and then it was blocked with TBS-Tween 0.1% and milk 5% for 1 hr at room temperature. The membrane was then washed for 10 min/3 times with the Tween-milk solution (now milk at 2.5%). The first antibody was added and left overnight at 4°C and at a dilution of 1:250. The membrane was washed for 10 min/5 times with the Tween-2.5% milk solution at room temperature. The second antibody-alkaline phosphatase was added for 1 hr at a dilution of 1:1000 at room temperature. The membrane was then washed for 10 min/5 times with the Tween-2.5% milk solution at 4°C. Finally, the membrane was treated with a Bio-Rad kit. The resulting bands were analyzed in a Kodak image station 440-CF using Kodak 1D 3.6 software. An antibody against β-actin was used as a control. Antibodies were obtained from Santa Cruz Biotechnology, Inc. Santa Cruz, CA.

### Determination of mRNA for AR at the ventral prostate

This experiment was performed using the semi-quantitative reverse transcription polymerase chain reaction procedure (RT-PCR) [23]. Tissue from the ventral prostate (100 mg) was homogenized in a Politron in 1 ml of Trizol, incubated for 5 min at room temperature, subjected to the addition of 200 μl of chloroform under continuous shaking, and incubated again for 3 min. The mixture was centrifuged (15 min at 8000 rpm, 8°C) and the liquid phase separated. Total RNA (RNAt) was precipitated with isopropilic alcohol (1:1 proportion), homogenized, incubated (10 min at room temperature), and centrifuged (10 min at 12000 rpm, 8°C). The resulting pellet was washed with 75% ethanol, and centrifuged (5 min at 7500 rpm, 8°C). The pellet was then dissolved with 50 μl of sterile water. 1 μl of this solution was added to 999 μl of sterile water (1:1000 dilution) to quantify RNAt. The absorbance was read at wavelengths of 260 and 280 nm with a spectrophotometer (Genova MK3).

Total cDNA (cDNAt) was obtained by thermocycling (3 min at 70°C) a mixture of 3 μl of RNAt, 1 μl of Oligo (dt), 1 μl of DNTP's, and 6 μl of sterile water. 9 μl of a cocktail containing 5 μl of Buffer 5x (50 mM Tris-HCl pH 8.3, and 40 mM KCl), 2 μl of DTT, 1 μl of RNAse inhibitor, and 1 μl of MMLV was then added. The mixture was incubated for 60 min at 37°C and then for 3 min at 94°C.

Amplification of cDNAt was accomplished with 2 μl of sense 5'-3' oligonucleotide (gtgtcgtctccggaaatgtt; corresponding to nucleotide 2781), 2 μl of antisense 3'-5' oligonucleotide (ggaatcaggctggttgttgt; corresponding to nucleotide 3030), 10 μl of cDNA, and 36 μl of a cocktail containing 1 μl of dNTP'S, 5 μl of Buffer 10X without MgCl_2_, and 1.5 μl of Mg^++ ^as MgCl_2 _(50 mM), 0.5 μl of Taq polymerase, and 28 μl of sterile water. The amplification was performed using the following steps: 1 cycle of denaturation (5 min at 94°C), 30 annealing cycles (1 min at 94°C), then 1 cycle at 60°C (1 min) and 1 at 72°C (2 min), and 1 elongation cycle (10 min at 72°C, then at 4°C). The resulting fragment consisted of 250 base pairs. The internal control consisted of the amplification of 228 base pairs, between nucleotides 471 and 698 of the gene for rat β-actin. The sequence used was sense 5'-3' (agcctagtacgtagcctacc; nucleotide 471) and antisense 3'-5' (ctctcagctgtggtggtgaa; nucleotide 698). Products were separated through a 1% agarose gel with 3 μl of Etide Bromure (10 mg/ml solution) for 30 min at room temperature and at 100 V. The resulting bands were analyzed in a Kodak image station 440-CF using Kodak 1D 3.6 software. Chemicals were obtained from Invitrogen, Mexico.

### Statistics

Although we were dealing with consecutive ejaculations, the data was derived from independent groups and our experimental design therefore precluded the use of repeated-measures paradigms. Hence, data were analyzed with a one-way ANOVA followed by a *post hoc *application of Dunnett's test when the F value showed a significant difference at p < 0.05. Statistical analyses were performed using the GB-STAT software package (Dynamic Microsystems, Inc., Silver Springs, MD), and significant differences were inferred when p < 0.05.

## Results

### Sexual behavior and prostate weight

**Figure 1:**
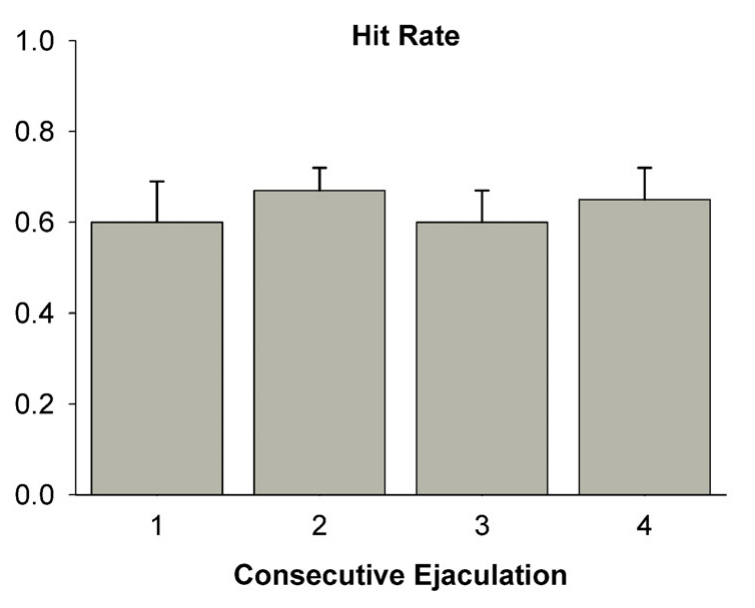
**The hit rate parameter**. The quantification of sexual behavior in male rats includes the recording of several parameters. Two of the most important are the frequency of mounts (FM) and frequency of intromissions (FI). The computation of FI/(FM+FI) results in a value representing the hit rate, which reflects the potency of males with respect to the execution of sexual behavior. Figure shows this parameter as measured for experimental males, and indicates that the execution of sexual behavior was stable across consecutive ejaculations and in the range acceptable for sexually experienced males.

**Figure 2:**
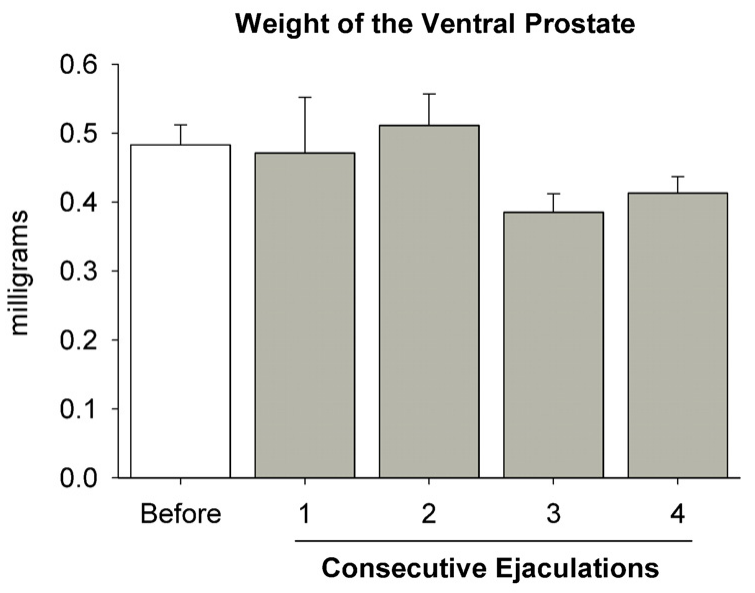
**Weight of the ventral prostate in sexually active male rats**. The graph shows that the weight of the ventral prostate was not affected by consecutive ejaculations, indicating that sexual experience and constant copulation did not impact its size.

Analysis of copulatory parameters showed that every male displayed standard ejaculatory patterns, as reported elsewhere [19], with 5–8 minutes of postejaculation refractory time. There were no significant differences for parameters between groups (Fig. 1 shows hit rate values). Furthermore, the constant execution of sexual behavior did not produce any change in the weight of the ventral prostate (Fig. 2).

### Serum levels of testosterone

**Figure 3:**
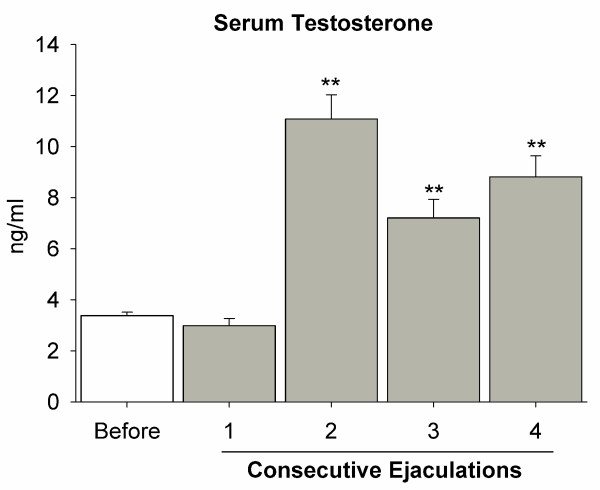
**Testosterone in serum after consecutive ejaculations**. The graph shows the basal level of testosterone in sexually experienced male rats before entering a sexual test (white bar). No effect was observed in this level after one ejaculation. However, the level increased to the highest value after the second and subsequent ejaculations. ** = p < 0.01.

Testosterone was detected in every plasma sample analyzed; it increased after two ejaculations and exhibited elevated levels even after four ejaculations (~10 ng/ml). Statistical analysis showed that overall testosterone levels were different, and *post hoc *results revealed significant increases after two, three and four ejaculations in comparison to the precopulatory value (p < 0.01). The lowest concentration of the hormone (~3.5 ng/ml) before sexual behavior represented the baseline level of testosterone, and was not modified by a single ejaculation (Fig. 3).

### Determination of AR at the ventral prostate

**Figure 4:**
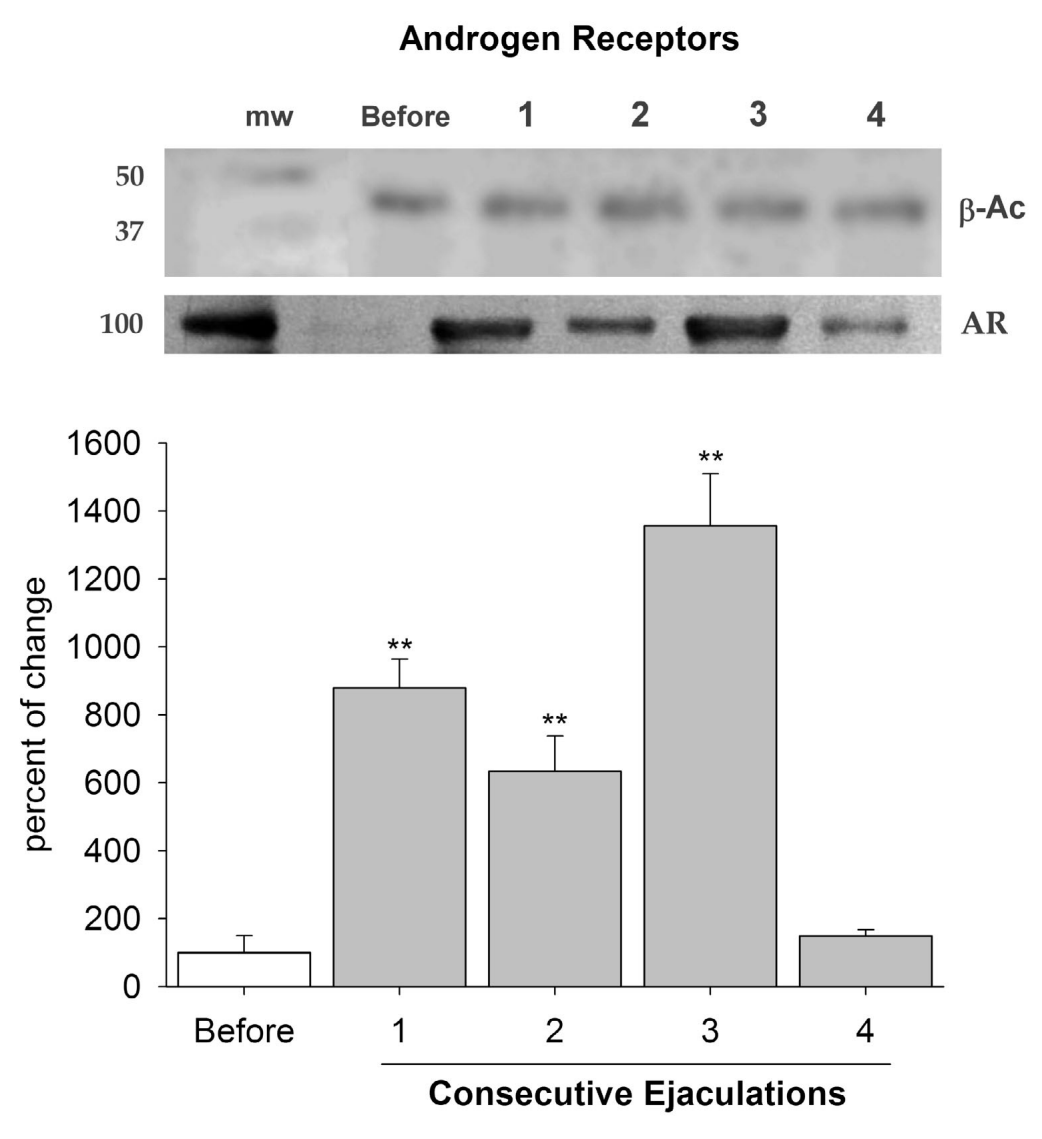
**Androgen receptors at the ventral prostate**. The execution of consecutive ejaculations had a strong impact on the level of androgen receptors at the ventral prostate. The graph shows that the protein for androgen receptors was detected in males that were not copulating (white bar). However, the concentration increased to its highest value after ejaculations one to three, and then returned to baseline levels after four ejaculations. Western blots at the top show bands for β-actin (β-Ac) and the androgen receptor (AR); mw = molecular weight; 1–4 = number of ejaculations. ** = p < 0.01.

The protein for AR was also detected in every sample analyzed. The concentration of AR was significantly higher in samples taken after one, two and three ejaculations in comparison to the precopulatory value (~800% increase; p < 0.01). However, the concentration of AR after four ejaculations was equal to that of baseline levels (Fig. 4).

### Determination of mRNA for AR at the ventral prostate

**Figure 5:**
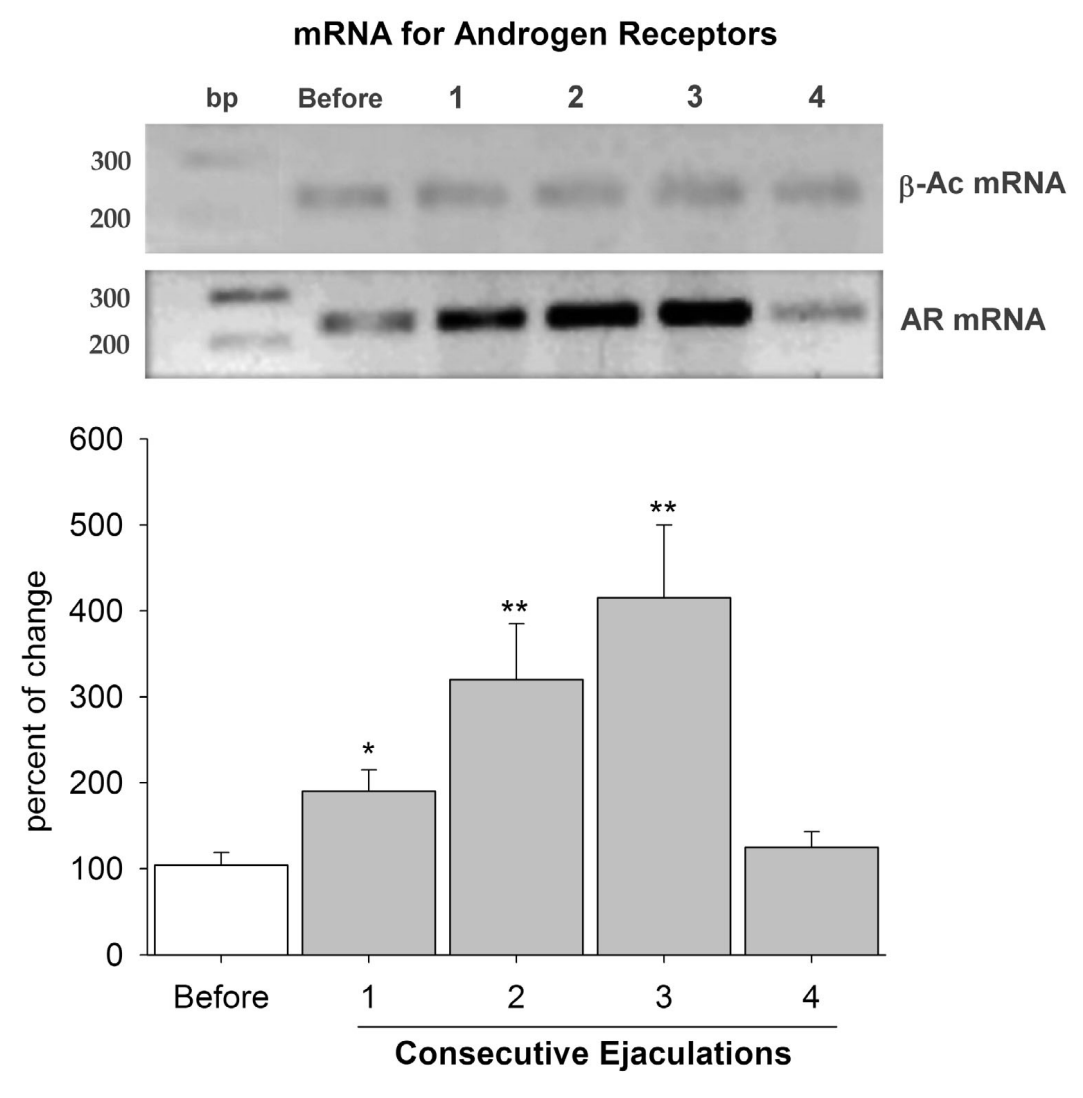
**mRNA for androgen receptors at the ventral prostate**. The level of mRNA for androgen receptors at the ventral prostate was modulated by copulation. The graph shows that mRNA was detected in males before sexual behavior (white bar). The level of mRNA increased constantly during the first three ejaculations, reaching the highest value after the third ejaculation. However, the concentration returned to baseline levels after four ejaculations. RT-PCR gels at the top show bands of mRNA for β-actin (β-Ac mRNA) and the androgen receptor (mRNA AR); bp = base pair; 1–4 = number of ejaculations. * = p < 0.05; ** = p < 0.01.

Levels of mRNA at the ventral prostate were determined for every sample, and increased significantly after one ejaculation (~100% increase; p < 0.05). Levels were highly significant after two and three ejaculations (p < 0.01), and kept increasing in these ejaculations (~200% and ~300% increase, respectively). However, the concentration of mRNA was equal to that of baseline levels after four ejaculations (Fig. 5).

## Discussion

The continuous execution of sexual behavior by experienced male rats did not affect the weight of the ventral prostate, but had important effects on the physiological responses of the gland. The faster copulation-induced response involved a significant increase in androgen receptors, observed after one ejaculation even before the higher expression of mRNA for androgen receptors or the serum elevation of the hormone. Thus, although the regulation of androgen receptors by its ligand has been reported by several authors [18,24], here we observed an increase of the receptor that precedes the increased serum levels of testosterone. This finding suggests another fast-activated mechanism for the rapid increase in the levels of androgen receptors at the ventral prostate, which could rely on the nerves supplying this region. Attached to the prostate is the major pelvic ganglion that receives two autonomic inputs from the spinal cord, the pelvic and hypogastric nerves, and gives off several branches that regulate the physiology of micturition and reproduction at the inferior genitourinary tract, representing preganglionic nerves activated during copulation [5,25]. These nerves are considered key components for the physiology of sexual glands, and the prostate responds to their autonomic neurotransmitters in order to accomplish its functions [26]. In spinal-cord injured males, an altered expression of androgen receptors and its mRNA has been reported in the prostate due to the lack of appropriate neural control [27]. It has also been discovered that some neuroendocrine peptides from nerve terminals control the prostatic response to testosterone [28]. All of these findings support our proposal that the rapid elevation of androgen receptors after one ejaculation seems to be modulated by nerve activity. Nevertheless, this issue still deserves further research.

The elevation of androgen receptors remained high after three ejaculations, but after four ejaculations it returned to baseline levels. This process was reliably quantified in every male of the experimental group. Thus, the constant execution of sexual behavior produced a sharp biphasic response in the concentration of androgen receptors at the ventral prostate, such that a sudden elevation of receptors after one ejaculation was followed by a sudden withdrawal after four ejaculations. The elevation was discussed above as a result of nerve activity, and the withdrawal could also be attributed to nerve control due to the fast removal of receptors. In order to account for the drop in the concentration of androgen receptors, it is important to consider its degradation. As several authors have pointed out, the ubiquitin-proteasome pathway is a complex cascade of enzymatic activity responsible for the proteolysis of proteins in which the androgen receptor is included [29-32]; hence, it is suggested that this is the active process for the copulation-induced degradation of androgen receptors at the ventral prostate. Although it has not been tested for the prostate gland, it is known that denervation of striated muscles activates the proteasome system in a manner that cannot be reversed by testosterone treatment [33], suggesting neural control of the proteasome pathway. Thus, a role of the autonomic nervous system in regulating this function in prostatic cells seems plausible.

The increase in androgen receptors at the ventral prostate is the first elevated response induced by copulation. At the same time, a gradual increase in the androgen receptor mRNA (AR-mRNA) commenced after one ejaculation, further increased after two, and reached a peak after three ejaculations. As in the case of the protein, the AR-mRNA level then experienced a sudden drop after four ejaculations. Thus, AR-mRNA also displayed a biphasic response, with a particular slope during its increase. Given that the nerve supply could also trigger the beginning of the AR-mRNA increase [27], the next question concerns the possible mechanism that sustains its continuous elevation. Some authors have shown that androgen stimulation down-regulates AR-mRNA [34], but here we saw that the maximum elevation of serum testosterone after two or three ejaculations did not arrest the continuous increase of AR-mRNA. Hence, we suggest that sexual behavior activates another prostatic process associated with the elevation of AR-mRNA in the presence of androgens. Data from control subjects showed a level of AR-mRNA indicating a basal gene transcription, and several reports have showed that post-transcriptional stabilization of AR-mRNA is a key process for controlling its metabolism [24,34,35]. Thus, it seems that a balance exists between gene transcription and stabilization that allows a basal/control level of AR-mRNA. Our proposal is that the execution of behavior modifies this balance by increasing the stabilization process, which reduces degradation and thereby induces a progressive accumulation of AR-mRNA due to the continuous basal gene transcription. Stability results after the binding of some specific proteins to the corresponding elements of mRNA, as represented by the AU-rich element binding protein or the Hu family of proteins [36-38]. During the fourth ejaculatory series, a rapid degradation process then occurs perhaps by the rapid removal of the binding proteins, which results in a fast destabilization of AR-mRNA. All of these processes support the hypothesis that the main control of AR-mRNA expression is post-transcriptional, and in this study we saw that it was modulated by behavior.

Serum levels of testosterone during mating were detected after two ejaculations and remained high for the rest of the experimental ejaculatory series. Though the elevation of testosterone in response to sexual stimuli in male rats was reported several years ago [39], in this study we showed an elevation during four consecutive ejaculations. This finding supports a number of reports showing the importance of testosterone in maintaining male reproductive physiology [20]. Here it is suggested that the basal circadian levels of serum testosterone [40] are optimal for triggering the first ejaculation, which in turn activates the system to release a higher concentration of the hormone to maintain the requirements of further ejaculatory events. Thus, a steady elevated level of serum testosterone after two ejaculations seems necessary to maintain the peculiar pattern of sexual behavior of male rats and the function of the prostate gland, among other processes. An important observation is the peak elevation recorded after the second ejaculation, because we reported previously a peak level of serum prolactin after the second ejaculation in male rats [1]. It therefore appears that in the complex pattern of male rat copulation, a second consecutive ejaculation triggers appropriate endocrine responses to ensure the reproductive potency of the couple.

## Conclusion

Four consecutive ejaculations by sexually experienced male rats had important effects on the physiological responses of the ventral prostate. Fast responses were induced as a result of sexual behavior that involved an increase and decrease in androgen receptors after one and four ejaculations, respectively. However, a progressive response was observed in the elevation of mRNA for androgen receptors, which also showed a fast decrease after four ejaculations. All of these changes with the prostate gland occurred in the presence of a sustained elevation of testosterone in serum that started after two ejaculations. A consideration of the fast-induced changes suggests that the nerve supply plays a key role in prostate physiology during the sexual behavior of male rats.

## Competing interests

The author(s) declare that they have no competing interests.

## Authors' contributions

MEH is in charge of this research, whose laboratory specializes in prostate research, and designed the project in collaboration with JM, who has solid experience in projects involving the use of sexual behavior in male rats; both researchers were in charge of organizing this manuscript. ASC was in charge of procedures to determine the serum level of testosterone, while GEAA and RD were in charge of procedures to determine androgen receptors. GEAA and FR were in charge of procedures to determine the androgen receptor mRNA. LIG and RT were responsible for behavioral observations and the management of animals.
